# Segmental artery clamping versus main renal artery clamping in nephron-sparing surgery: updated meta-analysis

**DOI:** 10.1186/s12957-020-01990-w

**Published:** 2020-08-16

**Authors:** Jinhong Xu, Shuxiong Xu, Biao Yao, Run Xu, Yuangao Xu, Fa Sun, Qian Qiu, Hua Shi

**Affiliations:** 1 Tongren City People’s Hospital Affiliated to Guizhou Medical University, Tongren, 554319 Guizhou China; 2 Department of Urology, Guizhou Provincial People’s Hospital Affiliated to Guizhou Medical University, Guiyang, 550002 Guizhou China; 3 Department of Oncology, Tongren City People’s Hospital Affiliated to Guizhou Medical University, Tongren, 554319 Guizhou China; 4 Institute of Tuberculosis Research, Chongqing Public Health Medical Center, Chongqing, 100036 China

**Keywords:** Renal cell carcinoma, Segmental arterial clamping, Main arterial clamping, Meta-analysis

## Abstract

**Objectives:**

Ischemia–reperfusion injury is harmful in partial nephrectomy (PN) in renal cell carcinoma. Choosing an appropriate surgical method is important to reduce ischemia–reperfusion injury. This study aimed to compare the effect of segmental artery clamping (SAC) and main renal artery clamping (MAC) on patients who underwent PN.

**Methods:**

Studies from January 2008 to November 2019 were identified by an electronic search of English and Chinese databases, including PubMed, Excerpt Medica Database, Cochrane Library, Wanfang, VIP, and Chinese National Knowledge Internet, without language restriction. Two reviewers were involved in the trial. The effects on operation time (OT), warm ischemia time (WIT), length of hospital stay (LOS), blood transfusion rate, postoperative complication rate, Clavien classification (≥ 3), and positive surgery margin (PSM) were evaluated using Stata software. Standardized mean difference (SMD, for continuous data) and pooled odds ratios (for count data) with 95% confidence interval (CI) were used as effect indicators.

**Results:**

Thirty-two studies were included. SAC decreased the 1-week (SMD = − 0.973; 95% CI = − 1.414, − 0.532; *P* = 0.000), 1-month (SMD = − 0.411; 95% CI = − 0.769, − 0.053; *P* = 0.025), and 3-month (affected kidney: SMD = − 0.914; 95% CI = − 1.662, − 0.617; *P* = 0.000) percentages of postoperative changes in renal function (estimated glomerular filtration rate) between the SAC and MAC groups. Sub-group analysis showed that the SAC group had longer OT (SMD = 0.562; 95% CI = 0.252, 0.871; *P* = 0.000) than the MAC group. However, no differences were observed in the OT, WIT, LOS, blood transfusion rate, postoperative complication rate, Clavien classification (≥ 3), and PSM between the two groups.

**Conclusions:**

SAC is superior to MAC in terms of short-term postoperative renal function recovery. The use of SAC or MAC depends on tumor size, location, surgical modality, and surgeon’s judgments.

## Introduction

Renal cell carcinoma (RCC) is the most lethal malignancy of urinary system tumors and has the second largest incidence with a higher incidence than prostate cancer and a slightly lower incidence than bladder cancer in North America and Europe [1]. The incidence of asymptomatic small renal cell carcinoma and micro renal cell carcinoma has remarkably increased with the increasing use of imaging for other medical indications by incidental detection [2].

Surgical resection was once the preferred treatment for renal tumors. Radical nephrectomy (RN) and partial nephrectomy (PN), which were first performed by Winfield in 1992, are the two main treatment options [3]. PN is appropriate in carefully selected patients with RCC and can reduce the risk of chronic kidney disease (CKD), decrease overall and tumor-specific mortality, and improve long-term renal function compared with RN [4–6]. However, renal artery occlusion is often required, and the accompanying thermal ischemic injury is an important cause of postoperative acute renal failure or long-term CKD [7]. Main renal artery occlusion is an extensive renal blood flow occlusion that blocks the blood supply of tumor and healthy nephrons, and the postoperative recovery of blood flow is bound to lead to the ischemia–reperfusion injury of healthy nephrons [8]. Blocking the renal artery branch that supplies tumor blood with highly selective blockade technology and not blocking the renal artery trunk are safe and feasible in PN, because PN can maintain the normal blood supply of residual kidney tissues and causes no remarkable difference in total renal function [9]. Trehan reported that off-clamp PN is associated with a considerably lower reduction in estimated glomerular filtration rate (eGFR) than on-clamp PN [10]. Robotic partial nephrectomy (RPN) with sequential segmental renal artery (SRA) clamping represents a good alternative for selective patients with multiple ipsilateral renal tumors. Precise segmental artery clamping (SAC) with the guidance of dual-source computed tomography (DSCT) can maximize renal function preservation [11]. Some studies in China reported that SAC is associated with decreased warm ischemia and appears promising in terms of preserving postoperative function [12, 13]. Segmental artery clamping (SAC) was preferred by many surgical doctors. Li and Zhang [12] showed that SAC is safer and has better renal function preservation compared with main artery clamping (MAC) in PN by reviewing the previous literature [13]. However, Taweemonkongsap showed that clamping techniques do not affect renal functions, and the complication rate is low even in a small volume center [14]. Therefore, the advantages and disadvantages of SAC and MAC lack systemic elaborations. The indicators for MAC and SAC are unclear. Therefore, tracking and analyzing current research for powerful and systematic evidence are necessary.

In this study, we conducted a retrieval and systematic analysis of the latest literature using case-control studies associated with comparing the features of SAC and MAC. Thus, this study can provide bases for evidence-based medicine to provide better services in clinical diagnosis and treatment.

## Methods

### Search strategy and selection criterion

This meta-analysis complied with the guidelines of the Preferred Reporting Items for Systematic Reviews and Meta-analyses. We selected relevant literature published from January 2008 to November 2019 by searching English and Chinese databases, including Cochrane Library, PubMed, Excerpt Medica Database, Web of Science, China National Knowledge Internet, VIP database, and WanFang database with the following text words and Medical Subject Heading (MeSH) terms: (Kidney neoplasms [MeSH] OR Renal tumor OR Kidney neoplasm OR Neoplasm, Kidney OR Renal neoplasms OR Neoplasm, Renal OR Neoplasms, Renal OR Renal neoplasm OR Neoplasms, Kidney OR Cancer of kidney OR Kidney cancers OR Renal cancer OR Cancer, Renal OR Cancers, Renal OR Renal cancers OR Cancer of the kidney OR Kidney cancer OR Cancer, Kidney OR Cancers, Kidney) AND (Partial nephrectomy OR Nephron sparing nephrectomy OR Nephron sparing surgery OR NSS) AND (Selective arterial clamping OR SAC OR Selective clamp OR Super-selective clamp OR Segmental artery clamp OR Zero ischemia) AND (Main arterial clamping OR MAC) AND (Comparative Study OR Comparative Studies [MeSH]). All eligible tests were considered for review regardless of language.

### Inclusion and exclusion criteria

The patients included were diagnosed with renal tumors, including benign and malignant tumors, and underwent SAC or MAC. Review literature and studies that were not control–case studies were not included in the present study. Documents without the necessary information or containing incomplete basic information were excluded. Low-quality studies were also excluded.

Two researchers (Xu and Zhang) reviewed the related articles separately and extracted the data using a uniform standardized table made with Excel 2010. The researchers preliminarily screened the article titles and abstracts independently. The studies that met the criteria were included in this meta-analysis. Any disagreements were resolved by consulting another researcher (SX Xu) until an agreement was reached. The targeted outcome measures were operating time (OT), estimated blood loss (EBL), warm ischemia time (WIT), blood transfusion rate, length of hospital stay (LOS), postoperative complication rate, postoperative eGFR change value, and percentage decrease in eGFR. In the process of data extraction, the continuous variables expressed using median and quartile spacing were converted to approximate mean and standard deviation, respectively, according to the methods proposed by Luo and Wan, respectively.

### Quality evaluation

The quality of each eligible article was assessed using the Newcastle–Ottawa Quality Assessment Scale (NOS). The papers with scores above 8 points were considered to have high quality. The papers with scores 6–8 points were considered methodologically sound. The studies scoring under 5 points were considered to have low quality and excluded from the final meta-analysis.

### Statistical analyses

Statistical analysis was performed with Stata software version 12.0 (2011) (Stata Corp, Colledge Station, TX, USA). The heterogeneity was assessed using the Cochrane’s *Q* test and the inconsistency index value (*I*^2^). *I*^2^ was more than 50%, which indicated that there was obvious heterogeneity. A random-effects model was used. A fixed-effects model was used when *I*^2^ was less than 50%. Otherwise, Descriptive evaluation was adopted for outcome indicators that cannot be quantitatively evaluated. *Egger*’s linear regression test was used to judge publication bias when the number of studies was more than 10.

## Results

### Baseline characteristics of the eligible studies

**Fig. 1:**
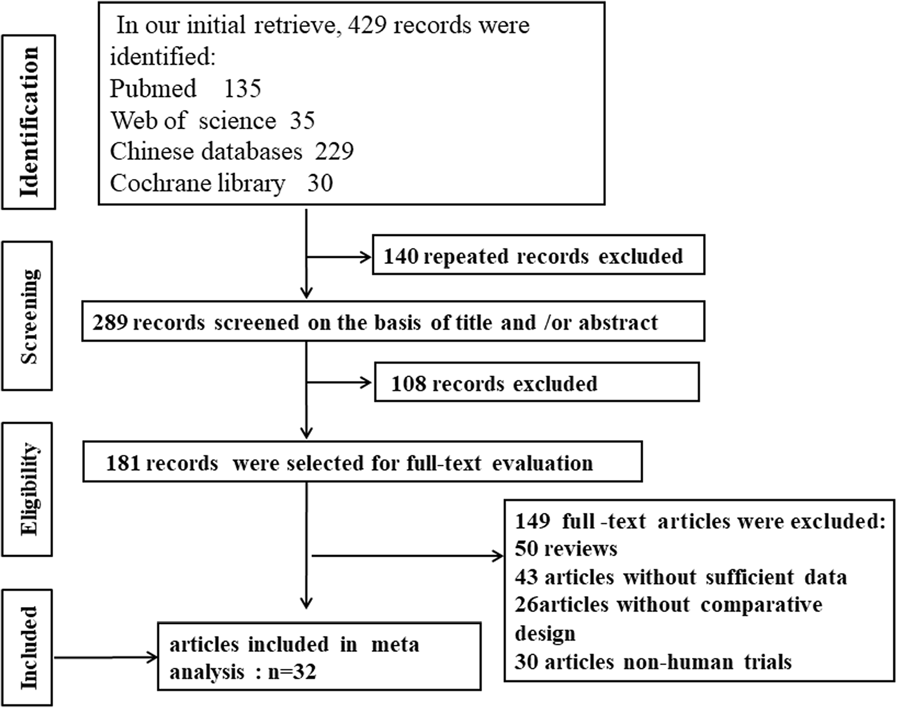
Flow chart of the included literature

A total of 429 studies were identified from a search of the aforementioned databases. Approximately 140 duplicate publications were eliminated after review. Finally, 32 studies, including 19 references in English and 13 references in Chinese, were included in this meta-analysis. These studies had a cumulative sample size of 3098, including 1289 cases in the SAC group and 1809 cases in the MAC group. The flow chart of the literature research phase is shown in Fig. 1. In terms of NOS scores, nine articles had six marks, 11 articles had seven marks, and 12 articles had eight marks. The basic information and NOS scores of the included literature are shown in Table 1. All data retrieved from the reviewed studies and recorded in an electronic database were as follows: demographic characteristics (age, sex, and body mass index), preoperative eGFR, tumor size (maximum diameter), and RENAL marks. The demographic data are shown in Table 2. No significant difference in demographic data was observed between the two groups.

**Table 1:** Basic information and remarks of the included papers

Study	Year	Type of study	Language	SAC	MAC	Operation methods	Observed indicators	Length of follow up	NOS marks	Overall conclusion in SAC compared with MAC
Daniele Mattevi	2018	Case-control	English	42	15	RPN	①②③④⑦⑧	1Mos	8	Equivalent ①②③④⑦⑧
Tawatchai Taweemonkongsap	2018	Case-control	English	38	27	RPN	①②③④⑤	18.2Mos	8	Equivalent ①②③④⑤, less number of patients with prolonged WIT
Gang Xu	2018	Case-control	Chinese	25	31	LPN	①②③⑥	3Mos	8	Equivalent ①②③⑥, superior postoperative renal function
Pengtao Wei	2018	Case-control	Chinese	29	36	LPN	①②③⑤⑦	3Mos	7	Equivalent ②③⑤⑦, more①, superior postoperative renal function
Yongjian Zhang	2018	Case-control	Chinese	28	22	LPN	①②⑤	3days	7	More ①③, equivalent ②, superior postoperative renal function
DeZhu Qi	2017	Case-control	Chinese	31	32	LPN	①②④⑤⑥	12Mos	8	Equivalent ②④⑤⑥, more①, superior postoperative renal function
Qian Cai	2017	Randomized controlled trial	Chinese	15	19	LPN	①②⑤	6Mos	8	Equivalent ②, more ①③, postoperative renal function
Paulucci	2016	Propensity-score analysis	English	66	132	RPN	①②③⑤⑥⑦	24Mos	7	Equivalent ①②③⑤⑥⑦
Pu Li	2016		English	314	152	LPN	①②⑥	70Mos	8	Equivalent ①②⑥, postoperative renal function
Furukawa	2016	Case-control	English	19	20	RPN	①②⑤⑧	1Mo	7	Equivalent ①②⑤⑧, postoperative renal function
Komninos	2015	Case-control	English	25	114	RPN	①④⑤⑥⑦	47Mos	8	Equivalent ①④⑤⑥⑦, superior postoperative renal function
Shin	2015	Case-control	English	20	97	RPN	③⑤⑥⑦	3Mos	7	Equivalent ③⑤⑥⑦, postoperative renal function
Akca	2015	Case-control	English	111	468	RPN	①②③⑤⑥⑦	6Mos	7	Less ①, equivalent ②③⑤⑥⑦
Wu Wei	2015	Case-control	Chinese	39	43	LPN	②③⑤⑥⑦	42Mos	6	Equivalent②③⑤⑥⑦, postoperative renal function.
JianZhou Liu	2015	Case-control	Chinese	29	27	LPN	①②⑤⑦	36Mos	6	Equivalent ⑤⑦, more①②, superior postoperative renal function
Peng Li	2015	Case-control	Chinese	10	13	LPN	①④⑤⑥	3Mos	8	More ①④, equivalent⑤⑥, superior postoperative renal function
Yuan Ruan	2016	Case-control	Chinese	45	45	LPN	①②⑤	22.5Mos	7	Equivalent in ①⑤, less②, superior postoperative renal function
Hao Yang	2015	Case-control	Chinese	35	45	LPN	②	24Mos	6	Less ②, superior postoperative renal function
JianFeng Zhao	2015	Case-control	Chinese	21	21	LPN	①②③④⑤⑥⑦	Not mentioned	6	More ①, equivalent ②③④⑤⑥⑦, superior early renal function
LiYong Xing	2014	Case-control	Chinese	27	28	LPN	①⑤⑥	Not mentioned	6	Equivalent①⑤⑥
Harke	2014	Case-control	English	15	15	RPN	①②⑤⑥	8days	7	Equivalent ①②⑤⑥, superior postoperative renal function
McClintock	2014	Case-control	English	42	42	RPN	①②⑤⑦	3Mos	8	Equivalent ①②⑤⑦, superior postoperative renal function
Yue Gao	2014	Case-control	Chinese	21	38	LPN	②③⑤⑥⑦	12Mos	6	More①,equivalent ③⑤⑥⑦, superior postoperative renal function
SiMei Zhu	2014	Case-control	Chinese	13	44	LPN	①②⑤⑥⑦	6Mos	7	Equivalent ①⑤⑥⑦, more②, superior postoperative renal function
Desai	2014	Case-control	English	58	63	RPN	①③④⑤⑥⑦	6Mos	7	Longer①, more④, equivalent in ③⑤⑥⑦, superior renal function
Sheng Li	2014	Case-control	Chinese	18	38	LPN	②③④⑤⑦	4Mos	6	Equivalent ②③④⑤⑦, superior postoperative renal function
Borofsky	2013	Case-control	English	27	27	RPN	①③④⑤⑥	1Mos	8	Equivalent ①③④⑤⑥, superior postoperative renal function
Martin	2012	Case-control	English	13	32	RPN	①③⑤⑥	3Mos	7	Equivalent ①③⑤⑥
JianGang Gao	2012	Case-control	Chinese	42	37	LPN	①②③④⑤⑥⑦⑧	48Mos	6	More ①, less②, eqiuvalent ③, superior postoperative renal function
Ng	2012	Case-control	English	22	22	RPN	①②③④⑤⑥⑦⑧	2Mos	8	Equivalent①②③④⑤⑥⑦⑧.
Pengfei Shao	2011	Case-control	English	31	37	LPN	①②⑤⑥	33Mos	8	Equivalent ①②⑤⑥, superior early postoperative affected renal function
Nohara	2008	Case-control	English	18	27	Not mentioned	①②⑤	8Mos	6	Equivalent ①②⑤, superior postoperative renal function

Note: ① OT, ② WIT, ③ PSM, ④ blood transfusion rate, ⑤ EBL, ⑥ postoperative complications (Hemorrhage, hematuria, urine leak) ⑦ LOS, ⑧ Clavien classification (≥ 3)

**Table 2:** Meta-analysis of demographics of the patients of the included studies

Indicators	Number of studies	Number of SAC	Number of MAC	Heterogeneity analysis					
				*χ*^2^	df	*I*^2^	*P* value	OR/SMD (95CI)	*P* value
Male	22	1072	1541	157.90	21	86.70	0.31	− 0.41(− 0.39, 0.118)	0.27
BMI (kg/m^2^)	21	979	1441	122.30	20	83.60	0.00	− 0.71(− 0.41, 0.07)	0.16
RENAL scores	16	841	1268	80.40	15	81.30	0.00	0.15(− 0.09, 0.40)	0.21
Tumor size(cm)	22	1000	1504	157.98	21	86.70	0.00	− 0.14(− 0.40, 0.40)	0.29
Preoperative eGFR (mL/min/1.73 m^2^)	21	694	1364	0.00	20	0.00	1.00	0.0(− 0.105, 0.105)	1.00

### Sensitivity analysis

Sensitivity analysis (Table 3) showed that OT, EBL, percentage of postoperative change in eGFR (1 week, 1 month, 3 months, and 6 months), positive surgery margin (PSM), blood transfusion rate, Clavien classification (≥ 3), and postoperative complications (hemorrhage, hematuria, and urine leak) in the two models were not remarkably different. Therefore, the results regarding these parameters were stable and reliable. By contrast, the results on WIT and LOS were unreliable (Table 3).

**Table 3:** The pooled effect of outcome indicators

Outcome indicators	SMD/OR	95%CI	Z value	*P*值
Operation time				
LPN	1.17	0.75, 1.58	5.54	0.000
RPN	0	− 0.38, 0.38	0	0.911
Overall	0.56	0.25, 0.87	3.55	0.000
Warm ischemia time				
LPN	0.12	− 0.25, 0.49	0.63	0.530
RPN	− 0.07	− 0.42, 0.28	0.38	0.702
Overall	0.04	− 0.21, 0.28	0.3	0.761
Estimated blood loss				
LPN	0.65	0.35, 0.95	4.29	0.000
RPN	0.41	0.11, 0.71	2.7	0.007
Overall	0.55	0.34, 0.75	5.1	0.000
Length of hospital stay				
LPN	− 0.131	− 0.355, 0.092	1.15	0.317
RPN	0.152	− 0.019, 0.322	1.74	0.182
Overall	− 0.006	− 0.166, 0.154	0.08	0.060
1-week postoperative change percentage in eGFR	− 0.97	− 1.41, − 0.53	4.32	0.000
1-month postoperative change percentage in eGFR	− 0.41	− 0.77, − 0.05	2.25	0.025
3-month postoperative change percentage in eGFR	− 0.26	− 0.59, 0.07	1.54	0.124
6-month postoperative change percentage in eGFR	− 0.08	− 0.40, 0.56	0.33	0.741
Positive surgical margin	1.173	0.547, 2.517	0.41	0.908
Blood transfusion rate	1.065	0.610, 1.860	0.22	0.825
Clavien classification(≥ 3)	0.89	0.507, 1.564	0.4	0.686
Postoperative complications (hemorrhage, hematuria, urine leak )	0.816	0.601, 1.107	1.31	0.191

### Publication bias

Publication bias for OT, LOS, percentage of postoperative change in eGFR (1 week, 1 month, 3 months, and 6 months), PSM, blood transfusion rate, Clavien classification (≥ 3), and postoperative complications (hemorrhage, hematuria, and urine leak) was assessed by Egger linear regression. The *P* values of OT, EBL, and LOS were less than 0.05, which suggested the presence of publication bias. The *P* values of the other parameters were greater than 0.05, which indicated no possibility of publication bias. The results are shown in Table 4.

**Table 4:** Egger linear regression results of outcome indicators

Outcome indicators	*P* value of *Egger* test
Operation time	0.027
Warm ischaemia time	0.287
Estimated blood loss	0.012
Length of hospital stay	0.008
1-week postoperative change percentage in eGFR	0.457
1-month postoperative change percentage in eGFR	0.744
3-month postoperative change percentage in eGFR	0.126
6-month postoperative change percentage in eGFR	0.898
Positive surgical margin	0.827
Blood transfusion rate	0.953
Clavien classification (≥ 3)	0.176
Postoperative complications (hemorrhage, hematuria, urine leak )	0.314

#### OT

**Fig. 2:**
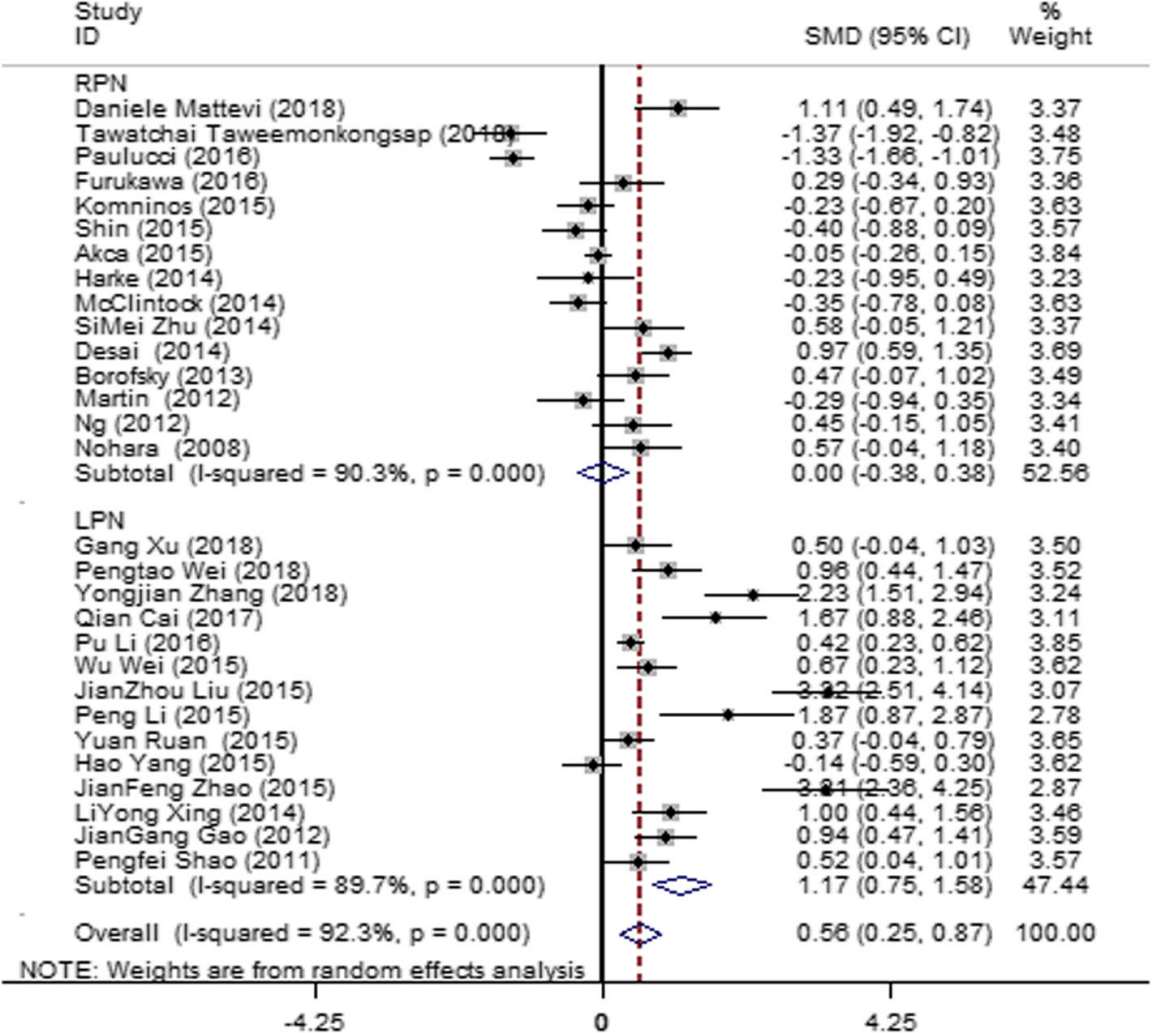
Forest plot and meta-analysis of the OT between the SAC and MAC groups

Thirty articles [12–41] were included in the literature. These studies showed heterogeneity (*I*^2^ = 92.3%, *P* = 0.000). Therefore, subgroup analysis was made according to different operation methods, namely laparoscopic partial nephrectomy (LPN) and robotic partial nephrectomy (RPN). Minimal heterogeneity was observed between the groups; thus, the operation method was not the main source of the heterogeneity. A random-effect model was used to pool the effectors. The result (Fig. 2) showed that OT was higher in the SAC group than in the MAC group (SMD = 0.56; 95% CI = 0.25, 0.87; *I*^2^ = 92.3%; *P* = 0.000), and no difference in OT was observed between SAC and MAC in the RPN group (SMD = 0.00; 95% CI = − 0.38, 0.38; *I*^2^ = 90.3%; *P* = 0.000).

#### WIT

**Fig. 3:**
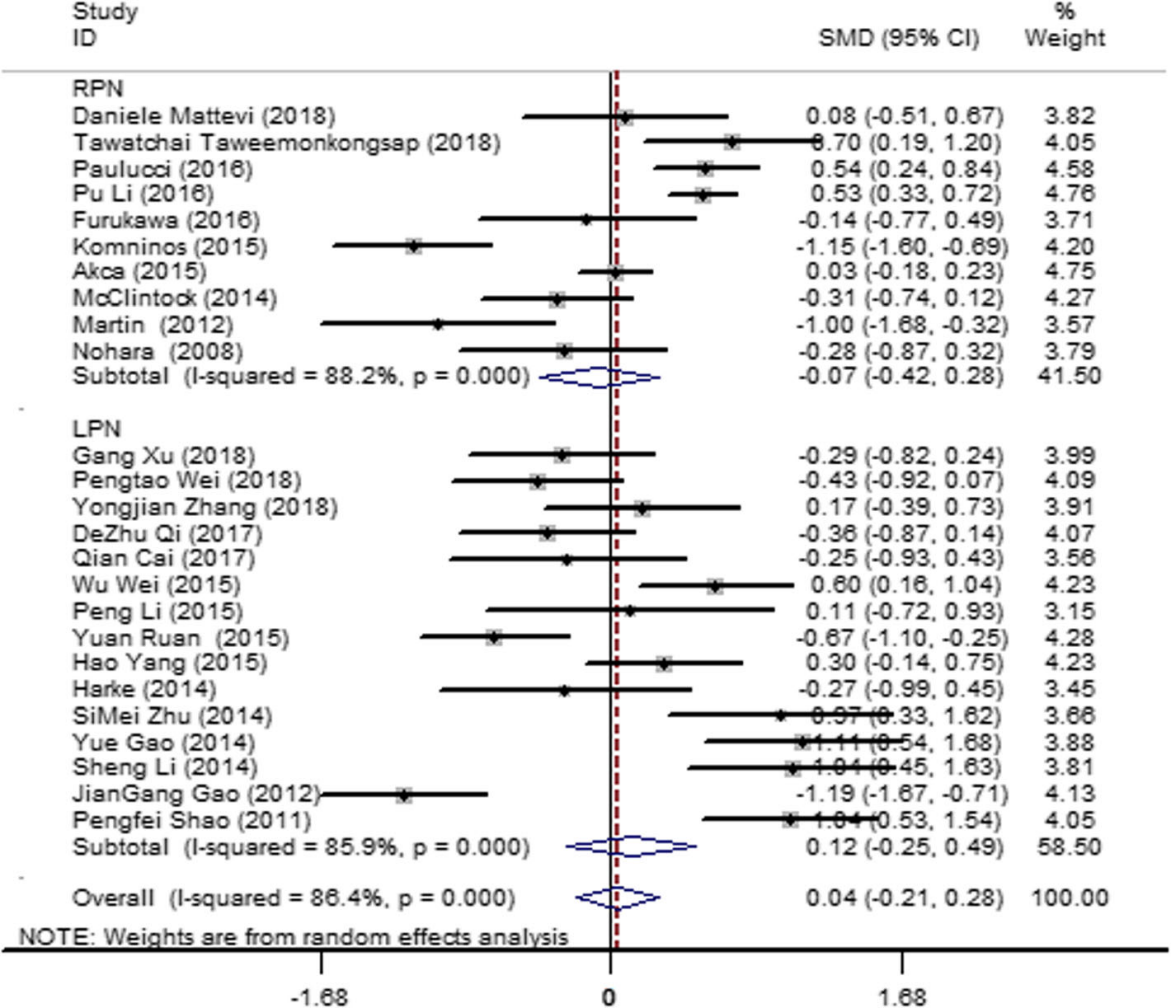
Forest plot and meta-analysis of the WIT between the SAC and MAC groups

Twenty-five articles [12–23, 26–29, 31–34, 37, 39, 42, 43] were included in the literature, and these studies showed heterogeneity (*I*^2^ = 86.4%, *P* = 0.000). Therefore, subgroup analysis was performed according to the different operation methods, LPN and RPN. Slight heterogeneity was found in the different groups, so the operation method was not the main source of heterogeneity. A random-effect model was used to pool the effectors, and the result (Fig. 3) showed no significant difference in the overall WIT between the SAC and MAC groups (SMD = 0.04; 95% CI = − 0.21, 0.28; *I*^2^ = 86.4%; *P* = 0.000).

#### EBL

**Fig. 4:**
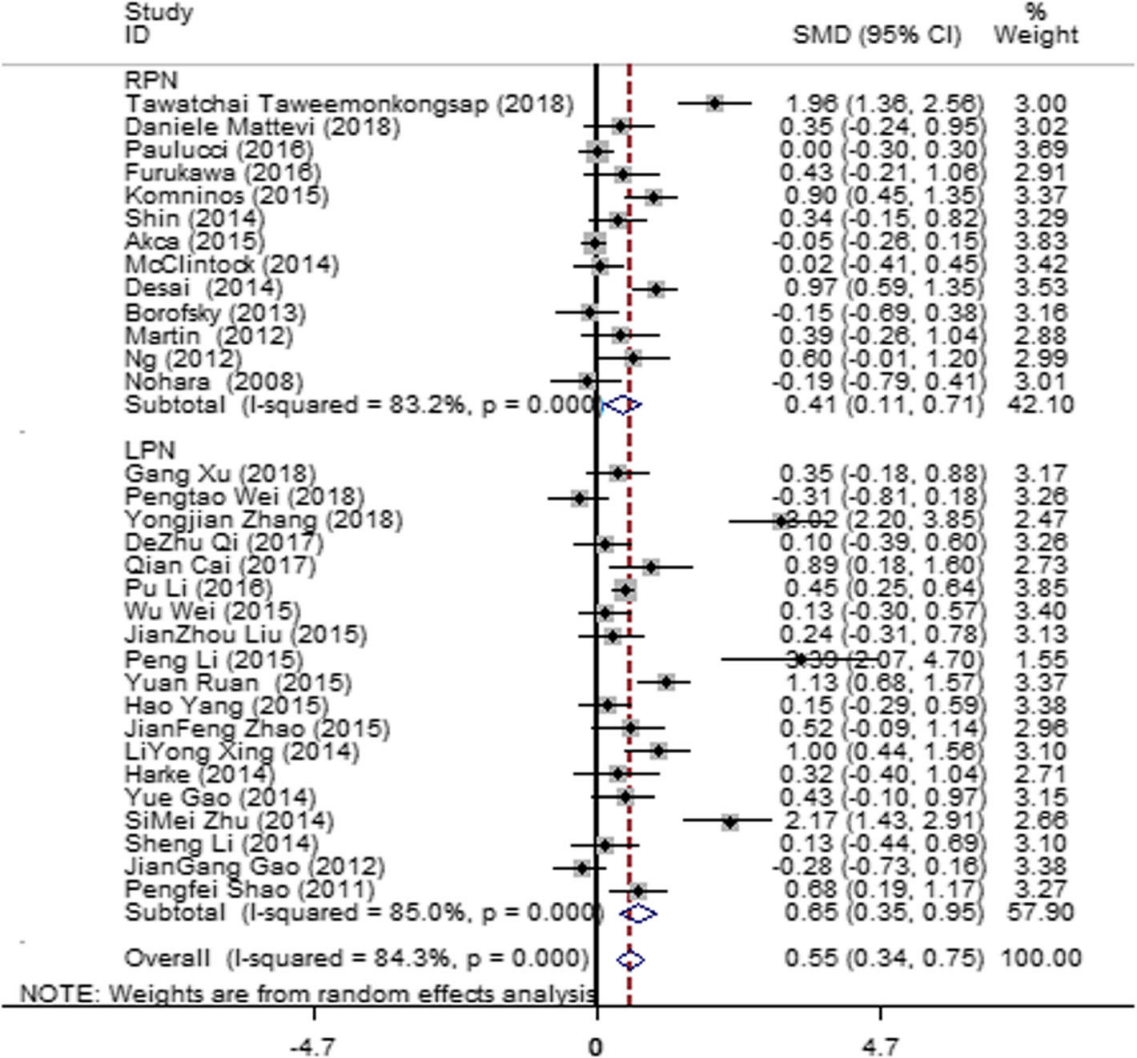
Forest plot and meta-analysis of the EBL between the SAC and MAC groups

Thirty articles [12–40, 43, 44] were included, and these studies showed severe heterogeneity (*I*^2^ = 84.3%, *P* = 0.000). Therefore, subgroup analysis was made according to the different operation methods, LPN and RPN. The heterogeneity in the different groups changed slightly; thus, the operation method was not the main source of heterogeneity. A random-effect model was then used to pool the effectors. The result (Fig. 4) showed that EBL was higher in the SAC group than in the MAC group (SMD = 0.55; 95% CI = 0.34, 0.75; *I*^2^ = 84.3%; *P* = 0.000).

#### Blood transfusion rate

**Fig. 5:**
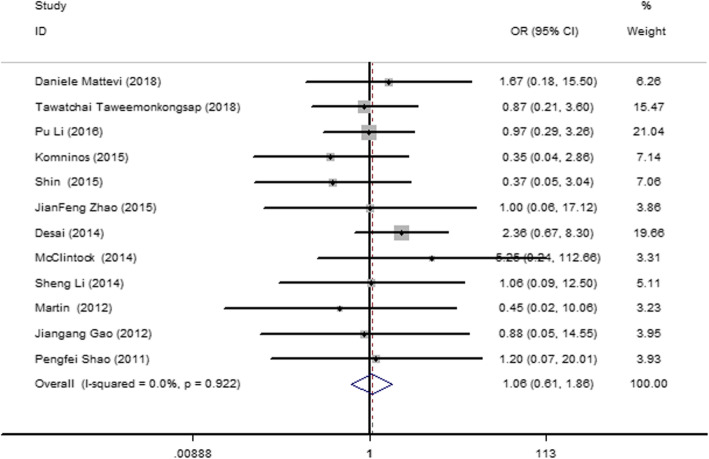
Forest plot and meta-analysis of the blood transfusion rate between the SAC and MAC groups

Twelve articles [12–14, 24, 26, 29, 30, 33, 35, 37, 43, 44] were included, and they showed no heterogeneity (*I*^2^ = 0, *P* = 0.922). Therefore, a fixed-effect model was used. The results (Fig. 5) revealed no significant difference in the blood transfusion rate between the SAC and MAC groups (odds ratio [OR] = 1.065; 95% CI = 0.610, 1.860; *I*^2^ = 0; *P* = 0.922).

#### LOS

**Fig. 6:**
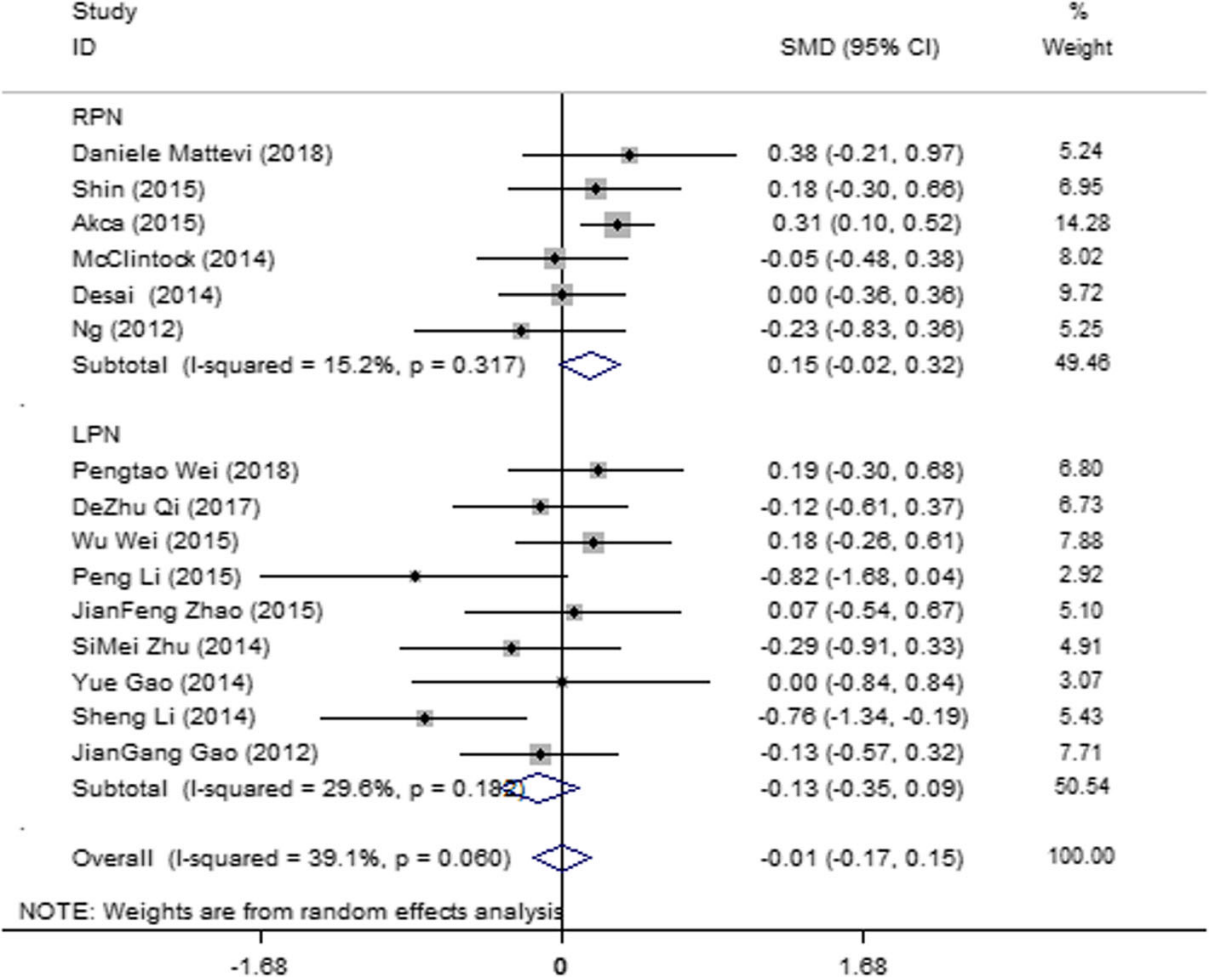
Forest plot and meta-analysis of the LOS between the SAC and MAC groups

Fifteen articles [16, 18, 20, 24, 26, 30, 31, 33–35, 38, 42, 43] were included, and they showed mild heterogeneity (*I*^2^ = 39.1%, *P* = 0.060). Subgroup analysis was performed according to the operation methods (LPN and RPN). *I*^2^ value was reduced to some extent in the subgroups; therefore, the operation method was partly the source of the heterogeneity. The results (Fig. 6) showed no significant difference in the LOS between SAC and MAC in the LPN (SMD = − 0.006; 95% CI = − 0.166, 0.154; *I*^2^ = 39.1%; *P* = 0.060) and RPN groups (SMD = 0.152; 95% CI = − 0.019, 0.322; *I*^2^ = 15.2; *P* = 0.317).

#### Postoperative complications (hemorrhage, hematuria, and urine leak)

**Fig. 7:**
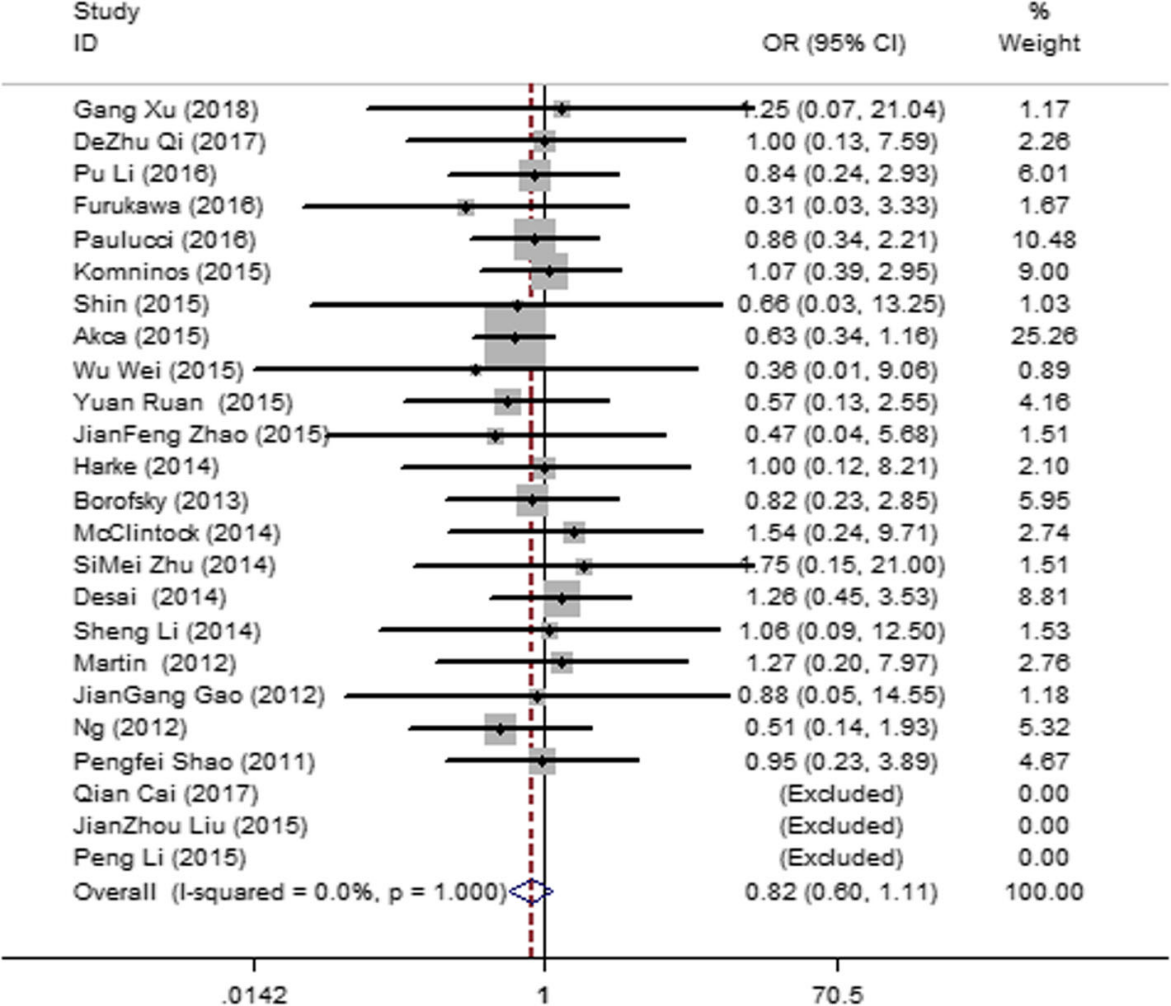
Forest plot and meta-analysis of the postoperative complication rate between the SAC and MAC groups

Twenty-four articles [12, 13, 15, 18–22, 24, 26–35, 37, 38, 42–45] were included, and they had no heterogeneity (*I*^2^ = 0, *P* = 1.00). Therefore, a fixed-effect model was used. The result (Fig. 7) showed no significant difference in the postoperative complications between the SAC and MAC groups (OR = 0.82; 95% CI = 0.60, 1.11; *P* = 0.191).

#### Clavien classification (≥ 3)

**Fig. 8:**
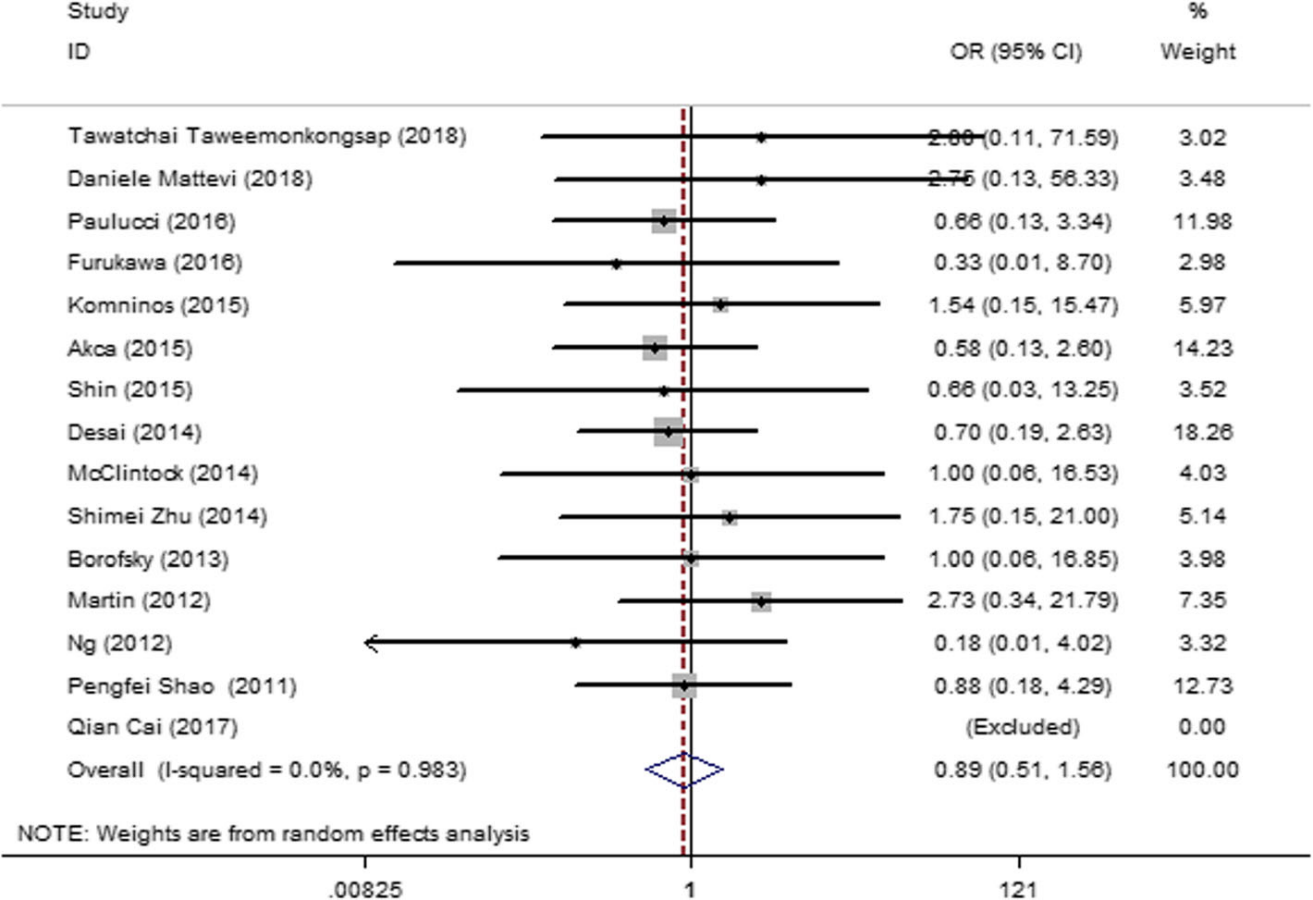
Forest plot and meta-analysis of the Clavien classification (≥ 3) between the SAC and MAC groups

Fifteen articles [13, 14, 19, 27–31, 33–35, 37, 38, 44, 45] were included, and no heterogeneity was found among the studies (*I*^2^ = 0, *P* = 0.983). Therefore, a fixed-effect model was used. The result (Fig. 8) showed no significant difference in the Clavien classification between the SAC and MAC groups (OR = 0.89; 95% CI = 0.51, 1.56; *I*^2^ = 0; *P* = 0.983).

#### PSM

**Fig. 9:**
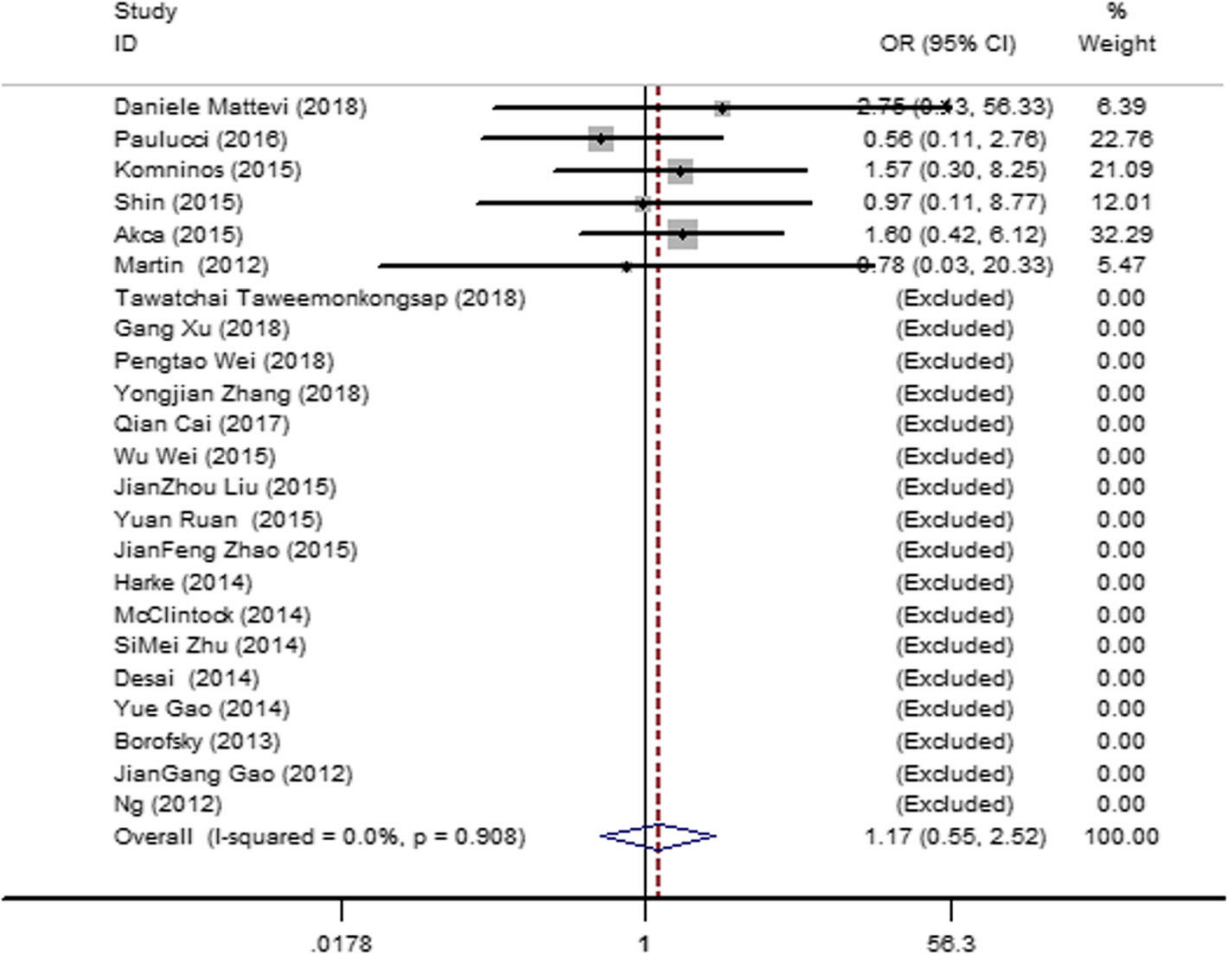
Forest plot and meta-analysis of the PSM between the SAC and MAC groups

Twenty-three articles [14–17, 20–22, 24, 26, 27, 29–35, 37, 38, 43–45]were included. No heterogeneity was observed among the studies (*I*^2^ = 0, *P* = 0.908), so a fixed-effect model was used. The result (Fig. 9) showed no significant difference in the PSM between the SAC and MAC groups (OR = 1.17; 95% CI = 0.55, 2.52; *P* = 0.908).

#### One-week postoperative change percentage in eGFR

**Fig. 10:**
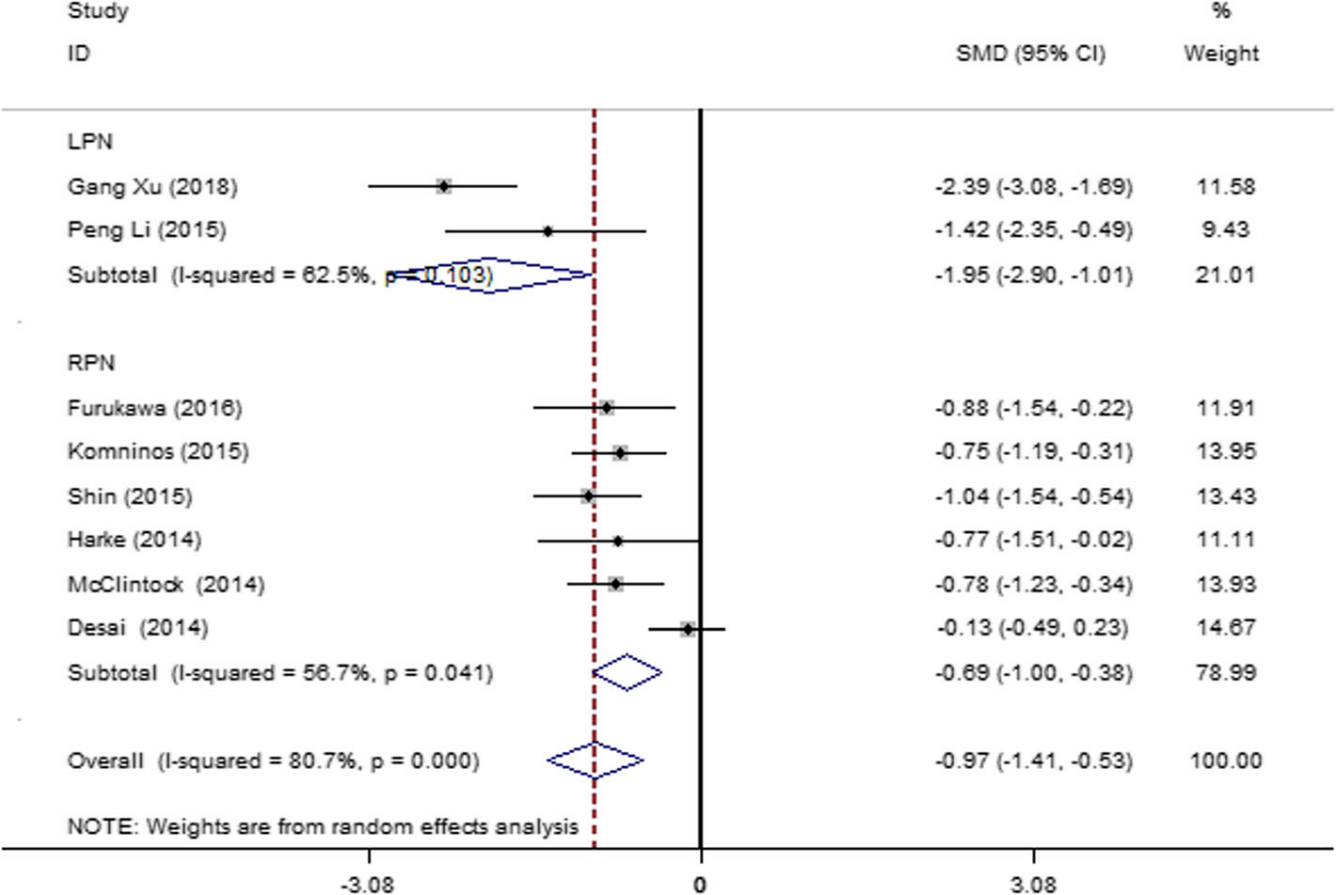
Forest plot and meta-analysis of the 1-week postoperative change percentage in the eGFR between the SAC and MAC groups

Eight articles [15, 28–30, 32, 33, 35, 42] were included, and severe heterogeneity was observed among the studies (*I*^2^ = 80.7%, *P* = 0.000). Subgroup analysis was performed according to the operation methods, LPN and RPN. *I*^2^ value was reduced to some extent in the subgroups; therefore, the operation method was partly the heterogeneity source. The result (Fig. 10) showed that the SAC group had a lower change in eGFR than the MAC group in the LPN (SMD = − 1.95; 95% CI = − 2.90, − 1.01; *I*^2^ = 62.5%; *P* = 0.103) and the RPN groups (SMD = − 0.69; 95% CI = − 1.00, − 0.38; *I*^2^ = 56.7%; *P* = 0.041) 1 week after the operation.

#### One-month postoperative change percentage in eGFR

**Fig. 11:**
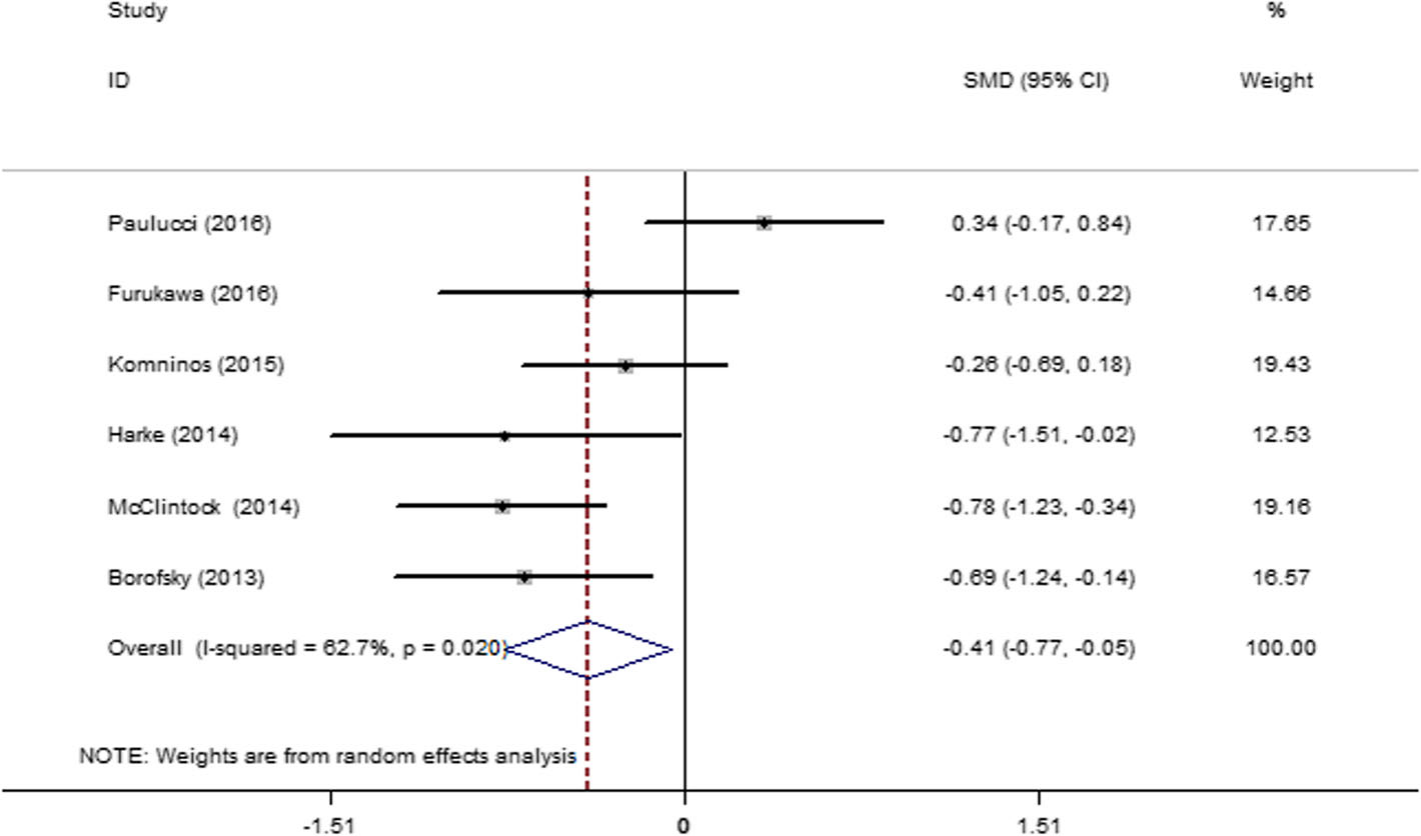
Forest plot and meta-analysis of the 1-month postoperative change percentage in the eGFR in the SAC and MAC groups

Six articles [27–29, 32, 33, 45] were included, and they showed moderate heterogeneity (*I*^2^ = 62.7%, *P* = 0.020). Therefore, a random-effect model was used. The result (Fig. 11) showed that the SAC group had a lower change in eGFR than the MAC group 1 month after the operation (SMD = − 0.41; 95% CI = − 0.77, − 0.05; *P* = 0.025).

#### Three-month postoperative change percentage in the total eGFR of both kidneys

**Fig. 12:**
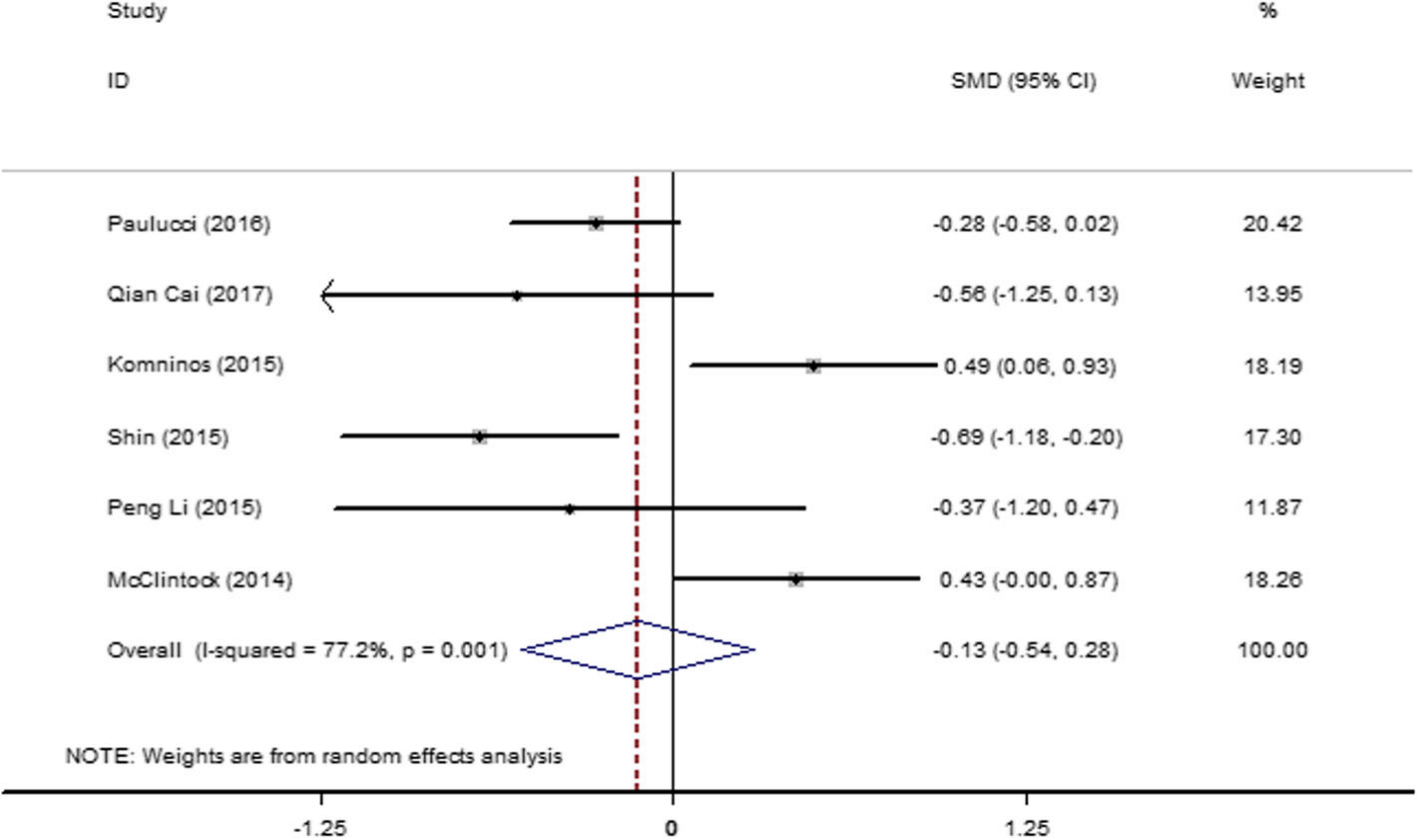
Forest plot and meta-analysis of the 3-month postoperative change percentage in the total eGFR between the SAC and MAC groups

Six articles [19, 27, 29, 30, 33, 42] were included and showed severe heterogeneity (*I*^2^ = 72.0%, *P* = 0.003). Subgroup analysis was performed according to the operation methods, LPN and RPN. *I*^2^ value was changed to some extent in the subgroups; therefore, the operation method was the source of heterogeneity in the LPN group but not in the RPN group. A random-effect model was used, and the result (Fig. 12) showed that the postoperative change percentages in the eGFR between the SAC and MAC groups at 3 months after the operation were not significantly different (SMD = − 0.35; 95% CI = − 0.72, 0.03).

#### Three-month postoperative change percentage in the eGFR of the affected kidney

**Fig. 13:**
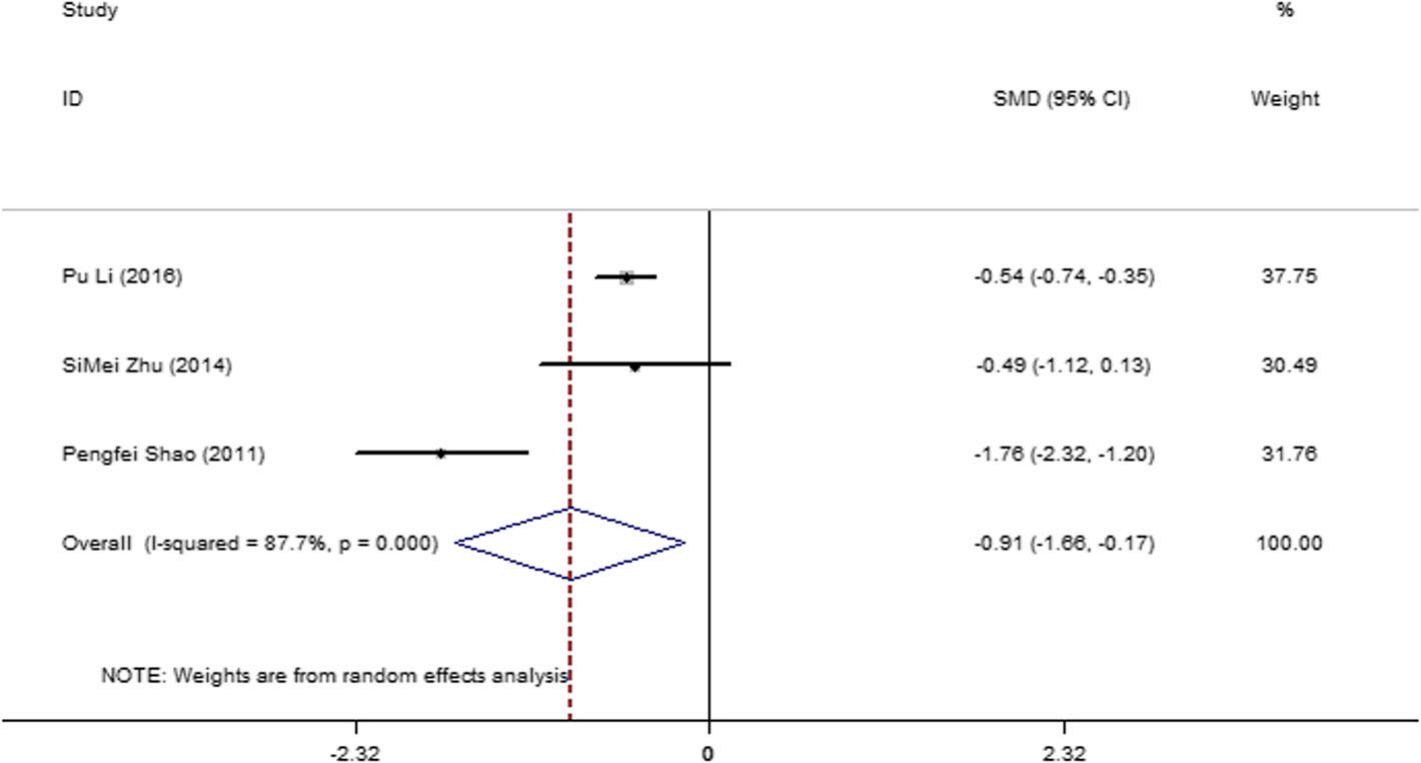
Forest plot and meta-analysis of the 3-month postoperative change percentage in the eGFR of the affected kidney between the SAC and MAC groups

Three articles [12, 13, 34] were compared, and they and they exhibited severe heterogeneity (*I*^2^ = 87.7%, *P* = 0.003). Therefore, a random-effect model was used. The result (Fig. 13) showed that the 3-month postoperative change percentage in the eGFR of the affected kidney was lower in the SAC group than in the MAC group (SMD = − 0.662; 95% CI = − 0.840, − 0.484; *P* = 0.000).

#### Six-month postoperative change percentage in the eGFR of both kidneys

**Fig. 14:**
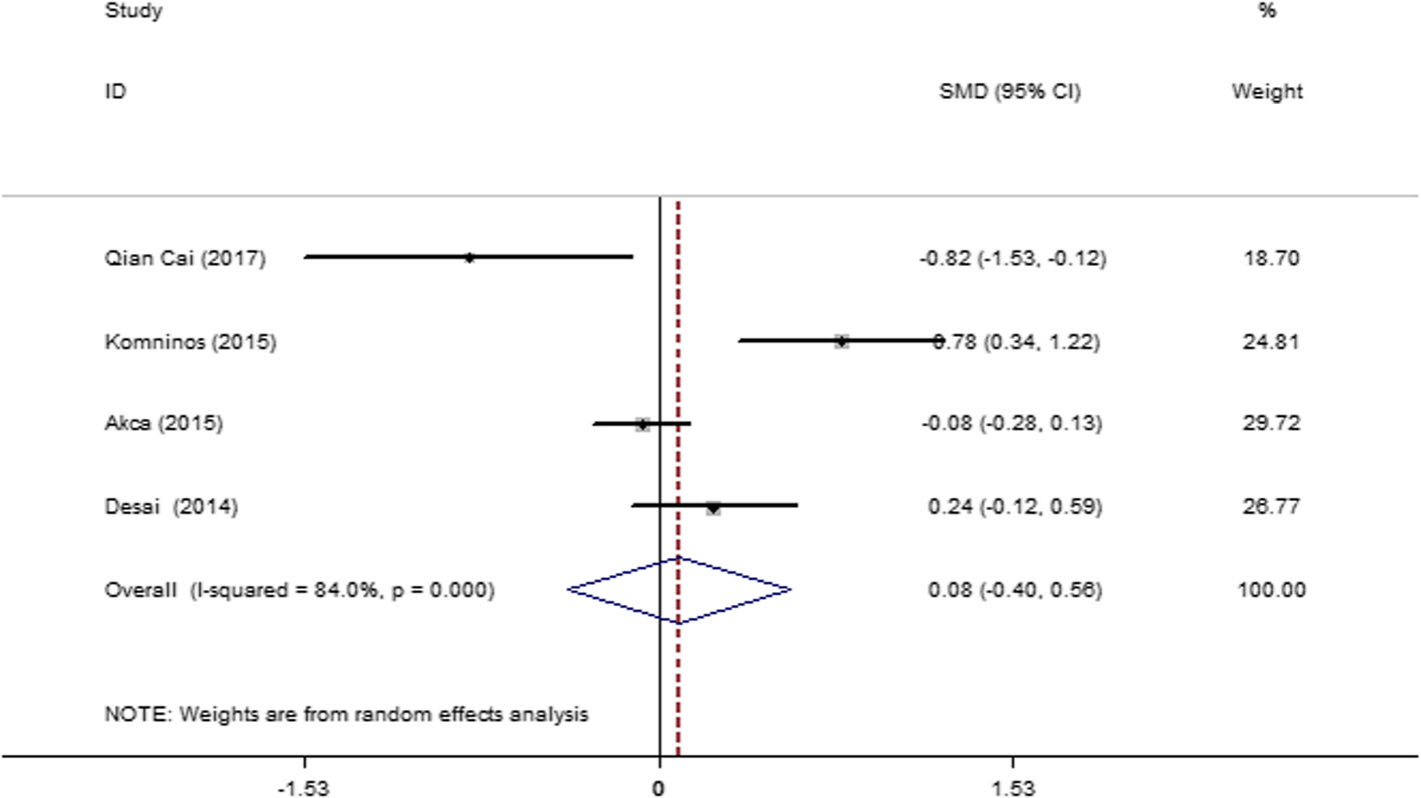
Forest plot and meta-analysis of the 6-month postoperative change percentage in the eGFR between the SAC and MAC groups

Four articles [19, 29, 31, 35] were included, and the articles had severe heterogeneity (*I*^2^ = 84.0%, *P* = 0.000). Therefore, a random-effect model was used. The result (Fig. 14) showed no significant difference in the 6-month postoperative change percentage in the eGFR between the SAC and MAC groups (SMD = 0.081; 95% CI = − 0.398, 0.560; *P* = 0.741).

## Discussion

In the current study, we systemically evaluated the effect of SAC and MAC on OT, EBL, WIT, LOS, postoperative complication rate, and postoperative percentage decrease in eGFR. The doctors conducted a case–control study by dividing the patients into the SAC and MAC groups. However, some patients included in the SAC group were subjected to MAC because of excessive bleeding from the defect, insufficient time to modulate the clamped branches, or no satisfactory ischemic area was obtained by clamping multiple branches. The two operations can be interchanged when necessary. Shao et al. first reported the nephrectomy of selectively blocking the renal artery and learned that SAC retains the affected nephrons and reduces the disadvantages of renal ischemic injury [13]. RPN with SRA clamping under the guidance of DSCT and skilled robotic experience is feasible and can maximize renal function preservation [46]. However, large-scale multicenter clinical studies are still needed to further prove these results.

Our results showed that OT was slightly longer in the LPN with SAC group than in the LPN with MAC group, whereas no difference was observed in the RPN group. PN with SAC requires the separation of the anterior and posterior branches of the renal artery and segmental renal arteries compared with traditional PN with MAC. The kidney should be slightly cut open to separate the third- and fourth-grade renal artery branches when necessary. Doing so requires additional steps and prolongs surgery. The duration of vessel separation during surgery is related to the doctor’s skill and familiarity with the anatomical location of blood vessels. In addition to the duration of surgery, WIT was emphasized because it is a key factor that affects renal function.

Ischemia–reperfusion injury is a major cause of acute ischemic renal injury. Temporarily blocking the renal pedicle vessels is necessary during PN to control intraoperative hemorrhage and maintain clear vision, but this procedure can cause renal ischemia–reperfusion injury [47]. LPN is widely used in the surgical treatment of renal tumors because of its safe and effective minimally invasive features. LPN with MAC has a good operative field, but long-term heat ischemia will cause damage to renal function [48, 49]. Benway showed that branch occlusion remarkably reduces thermal ischemia–reperfusion injury in a pig kidney model [50]. Selective renal artery branch blocking technology has been gradually used in PN. The technology only blocks the renal artery branch that supplies blood to the tumor and can effectively avoid or reduce thermal ischemia injury in the normal renal unit. A WIT over 30 min will produce irreversible damage to kidney function. However, WIT is difficult to control in deeper positions, especially for cases in which the drainage system should be sutured. This meta-analysis showed that OT was longer in the SAC group than in the MAC group in LPN, but no significant difference was observed in the OT between the SAC and MAC groups in RPN. In addition, no significant difference was observed in the WIT between the SAC and MAC groups. LOS is affected by several factors, including a patient’s condition. Our study showed no obvious difference in LOS between PN with SAC and PN with MAC; thus, PN with SAC did not affect LOS.

The results showed that EBL was greater in the SAC group than in the MAC group (*P* = 0.000). However, no significant difference was observed in the blood transfusion rate between the SAC and MAC groups. The combined effect indicator showed a difference between the two groups, but the amount of bleeding is commonly associated with a doctor’s experience and skill. The kind of operation adopted depends on the ability of the doctor and the patient’s condition. Seventeen of 23 articles showed that none of the patients had PSM. The remaining six articles showed no difference in PSM between the SAC and MAC groups. The combined effect indicator also proved that no difference was observed between the two groups (*P* = 0.8). Thus, no remarkable difference was found in the postoperative complications (hemorrhage, hematuria, and urine leak) and Clavien classifications (≥ 3) between the SAC and MAC groups. Heterogeneity analysis showed no heterogeneity among these studies.

The eight articles [15, 28–30, 32, 33, 35, 40] related to 1-week postoperative change percentage in eGFR, which were eligible for this meta-analysis, indicated that the SAC group had lower changes than the MAC group in LPN and RPN. The six articles [27–29, 32, 33, 36] related to the 1-month postoperative change percentage in eGFR indicated that the SAC group had lower changes than the MAC group. The six articles [19, 27, 29, 30, 33, 42] related to the 3-month postoperative change percentage in the total eGFR of both kidneys indicated that the SAC and MAC groups had no significant difference in postoperative change in eGFR after 3 months of operation. The three articles [12, 13, 34] related to the 3-month postoperative change percentage in the eGFR of the affected kidney showed severe heterogeneity and indicated that the SAC group had lower changes in eGFR than the MAC group after 3 months of operation. The four articles [19, 29, 31, 35] related to the 6-month postoperative change percentage in the total eGFR of both kidneys showed severe heterogeneity and indicated that the two groups had no significant difference in eGFR change after 6 months of operation.

The number of articles investigating eGFR change ranged from 3 to 10. Our study showed that SAC reduced the percentage change in eGFR within 3 months but had no obvious effect on eGFR in 6 months. An analysis of eGFR change beyond 6 months was not carried out because of the lack of related literature. No kidney function loss by 18 months (median) of follow up was observed in T Taweemonkongsap’s study. The present study suggested that SAC had a certain advantage in the short-term recovery of renal function, and this finding requires more random clinical experiments for verification. Our study results provide a basis for surgeons to actively screen and adapt patients to perform branch occlusion. Some patients might be good candidates for SAC. The patients with a tumor near the center of the renal hilum and those with large tumors may benefit from SAC. Komninos [29] suggested that patients who have tumors near the renal hilum, a solitary kidney, or preoperative renal damage are suitable for SAC. Desai [35] reported that interior, central, and bipolar tumors are suitable for SAC. Martin [37] indicated that SAC is easier to conduct on the right side because the right main renal artery is longer and offers more space for branch dissection than the left side.

## Deficiencies

A large number of literature have been searched according to the inclusion and exclusion criteria; however, most of the studies included in this paper were not (RCTs), and many of the data were expressed in different ways. Some data were calculated by transforming the median and quartile into mean and standard deviation, respectively. Different articles varied in the expression index of kidney function. Some studies used eGFR, whereas others used blood creatinine; therefore, merging the data was difficult. And the surgeon experience, volume, as well as the closure technique cannot be controlled in the analysis. Moreover, the research results showed publication bias in three indicators.

## Conclusion

The current result suggests that SAC is superior to MAC in terms of short-term postoperative renal function recovery. SAC is safe for selective patients when performed by a skilled surgeon. No clear differences in OT, WIT, LOS, blood transfusion rate, and postoperative complications were observed when compared with the alternative (i.e., SAC vs. MAC). However, RCTs are needed to support these preliminary findings.

## Abbreviations

CI: Confidence interval
SMD: Standard mean deviation
SAC: Segmental artery clamping
MAC: Main artery clamping
PN: Partial nephrectomy
PSM: Positive surgery margin
eGFR: Estimated glomerular filtration rate
OT: Operation time
WIT: Warm ischemia time
LOS: Length of hospital stay
